# Memory Deficits in Parkinson’s Disease Are Associated with Impaired Attentional Filtering and Memory Consolidation Processes

**DOI:** 10.3390/jcm12144594

**Published:** 2023-07-10

**Authors:** Eun-Young Lee

**Affiliations:** Department of Health Care and Science, Dong-A University, Busan 49315, Republic of Korea; enyoungee@dau.ac.kr

**Keywords:** Parkinson’s disease, short-term memory, episodic memory, attention, memory consolidation, temporal lobe, Alzheimer’s disease

## Abstract

The present study examined mechanisms underlying memory deficits in Parkinson’s disease (PD) and their associations with brain structural metrics. Nineteen PD patients and twenty-two matched controls underwent two memory experiments. In Experiment 1 (delayed memory task), subjects were asked to remember an array of colored rectangles with varying memory set sizes (Low-Load (2 items), Low-Load (relevant 2 items) with Distractor (irrelevant 3 items), and High-Load (5 items)). After a 7 s delay period, they reported whether the orientation of any relevant figures had changed (test period). In Experiment 2 (working memory task), memory arrays were presented in varying set sizes (2 to 6 items) without distractors, followed by a 2 s delay period and a subsequent test period. Brain MRI data were acquired to assess structural differences (volumes and cortical thickness) in areas related to attention, working memory storage capacity, and episodic memory. Multivariate analyses of covariance revealed that, compared with controls, PD patients had lower memory capacity scores in all memory load conditions for Experiment 1 (*p* < 0.021), whereas there were no group differences in any memory load conditions for Experiment 2 (*p* > 0.06). In addition, PD patients had lower cortical thickness in the left superior temporal gyrus (*p* = 0.02), a region related to the ventral attentional system. Moreover, regression analyses revealed that lower cortical thickness values in the left superior temporal gyrus significantly predicted lower memory scores of Low-Load and Low-Load with Distractor conditions in Experiment 1 (*p* < 0.044) and lower scores of memory load conditions of 4 and 5 items in Experiment 2 (*p* < 0.012). These findings suggest that memory deficits in PD may partly be due to impaired attentional filtering and memory consolidation processes that may be related to superior temporal neurodegeneration. Future studies are warranted to confirm the current findings to guide the development of effective treatments for memory deficits in PD.

## 1. Introduction

Parkinson’s disease (PD) is a progressive neurodegenerative disorder with predominant loss of dopaminergic neurons in substantia nigra pars compacta and subsequent depletion of dopamine levels in the basal ganglia. The prominent characteristics of PD include motor symptoms such as tremor, rigidity, and bradykinesia. While motor symptoms dominate clinical pictures in PD, many patients with PD experience a wide range of nonmotor symptoms. These may include autonomic disturbances (e.g., constipation and bladder control problems) and sensory complaints (e.g., numbness, burning, or tingling sensation) but also include psychiatric (e.g., depression and anxiety) or nonpsychiatric cognitive dysfunctions such as problems with executive functions, attention, and memory [1,2,3,4].

Given that the basal ganglia have extensive interconnections with the prefrontal cortex [5,6], cognitive symptoms in PD are often ascribed to compromised information flow through this frontostriatal pathway [5]. In fact, the pattern of cognitive deficits in PD appears to be similar to that observed in frontal lobe patients, such as difficulties with planning, selective attention, and set shifting [7,8,9]. Converging evidence, however, suggests that PD patients may also experience various cognitive dysfunctions beyond frontal-lobe-related executive dysfunctions [10,11]. 

### 1.1. Working Memory Deficits in PD

Working memory, previously known as short-term memory, has traditionally been considered as a capacity-limited storage system that maintains information over a period of seconds [12,13]. This passive information storage concept has, however, been criticized and replaced with a multicomponent theory by incorporating an information processing component into working memory [14,15,16]. Thus, working memory is currently termed as a capacity-limited system which temporarily maintains information in a highly active and accessible state but also manipulates that information for performing tasks [17,18,19,20]. Accordingly, poor performance on working memory tasks can be due to inability to maintain information in memory space (reduced storage capacity), inability to effectively process information during encoding and retrieval processes, or both [18,21]. Distributed cortico-striatal and frontoparietal attentional networks have been suggested to be involved in these two aspects of working memory, and related brain network dynamics may particularly be modulated by the dopaminergic system [20,22,23,24]. 

Working memory deficits are frequently observed in Parkinson’s disease, probably due to dopaminergic cell deaths in the basal ganglia that can affect interconnected cortical functions [25]. Previous studies, for example, reported that PD patients had poor performance on various working memory span tasks with both verbal and visuospatial materials [26,27]. The exact nature of working memory deficits in PD, however, remains clear. Some studies suggested that PD patients may suffer from reduced storage capacity [28] even down to the level of half the memory capacity of neurologically normal subjects [29]. Other studies, however, suggested that PD patients may have intact storage capacity but impaired processing component of selectively updating relevant information into working memory [30,31]. As a result, PD patients may unnecessarily usurp capacity-limited working memory space with irrelevant information, leading to poor accuracy for the relevant target information [28,30]. 

### 1.2. Episodic Memory Deficits in PD

Episodic memory is a system that involves consciously retrieving information (e.g., events) that was acquired in a particular time and space [32] and is known to be a key function of medial temporal lobe memory areas, especially the hippocampus [33,34,35]. Episodic memory problems have been reported as the earliest neurobehavioral deficits in Alzheimer’s disease (AD) [36], the most common age-related neurodegenerative disorder, comprising about 50–70% of dementia cases [37]. AD is characterized by accumulations of β-amyloid plaques and tau tangles in the brain, with the most prominent neuronal damage noted in the hippocampus [38]. Since the disease progresses relentlessly once AD-related clinical symptoms are manifested to the diagnostic level, AD-related early changes such as decline in episodic memory and medial temporal structural differences have been utilized for early detection of AD at-risk populations [39,40].

Although PD present difficulties in various cognitive domains, deficits in episodic memory [36] are one of the most common and devastating cognitive symptoms in PD [9,41,42,43]. Previous studies reported more than 20% of newly diagnosed PD patients had lower performance on episodic memory tasks [44]. Correspondingly, the prevalence rate of mild cognitive impairment (MCI), a risk factor for dementia development, and dementia cases in PD range from ~20 to 70% for MCI and ~20 to 50% for dementia [10,45]. As the disease progresses, up to 80% of PD patients may eventually develop dementia within 20 years of the disease [9,40,41]. The exact nature of episodic memory deficits in PD is, however, still unclear. Episodic memory deficits in PD, for example, have often been considered secondary to attentional or executive deficits resulting from dysfunctional cortico-striatal circuits. Accordingly, episodic memory problems in PD may mainly occur during information retrieval and to lesser extent during encoding, rather than during stabilizing newly learned information (memory consolidation), a function closely related to the hippocampus [46,47]. There is, however, also evidence suggesting that memory consolidation processes may also be impaired in PD [48,49].

### 1.3. Introduction to Current Hypotheses

In the present study, two visual array comparison experiments were conducted to examine the mechanisms underlying potential memory deficits in PD patients. In both experiments, participants viewed an array of colored rectangles. In some trials, the presented array contained task-irrelevant items that should be ignored. After a short (2 s) or relatively long (7 s) delay, subjects reported whether the orientation of any relevant figures had changed. Structural differences were assessed by volumes or cortical thickness in brain areas related to attention (e.g., superior frontal gyrus, superior parietal lobe, and intraparietal sulcus for the dorsal attentional system and inferior and middle frontal gyri, inferior parietal lobe, and superior temporal gyrus for ventral attentional system), working memory storage capacity (e.g., intraparietal sulcus), and episodic memory (e.g., medial temporal lobe structures). Our central hypotheses are as follows: (1) compared with controls, PD patients will show lower performance in both working memory and delayed memory tasks; (2) the memory performance in PD will be lower with distractors than without; (3) there will be significant structural differences in brain areas related to attention, working memory storage capacity, or episodic memory; and (4) there will be positive associations between MRI structural metrics in ROIs and memory metrics. 

## 2. Materials and Methods

### 2.1. Participants

Forty-one subjects (nineteen patients with Parkinson’s disease (PD) and twenty-two age- and education-matched neurologically normal subjects) were recruited from PD support group meetings and local community centers in Missouri, USA. All subjects except for one patient and one control subject reported having normal color vision and normal or corrected-to-normal acuity. One patient and one control who were identical twins were partially color-blind, but they were able to tell the difference between red and green rectangles used in this study. All subjects had Mini-Mental Status Examination (MMSE) scores ≥ 26. Depression was evaluated by means of Geriatric Depression Scale (GDS)—long form [50]. Three patients and one control subject were taking antidepressants (e.g., Fluoxetine, Bupropion, or Sertraline) at the time of the study. The general pattern of results was essentially the same with or without these three patients, so their data were retained. 

PD patients were free from other neurological disorders. Fifteen patients were receiving the dopamine precursor Levodopa as treatment. One patient was not taking any antiparkinsonian medication. Another patient was only taking Pramipexole (a dopamine agonist). The remaining two patients were receiving Ropinirole in conjunction with Trihexyphenidyl (an anticholinergic agent) or Azilect (an MAO inhibitor). On the morning of the experiment, PD patients skipped their initial dose of antiparkinsonian medication. The mean withdrawal period of 11 h (at least 9 h) would not be enough to achieve complete clearance; rather, it was intended to enhance differences between groups while minimizing the burden imposed on patients. The severity of the disease was reassessed just before the start of the experiment using the Hoehn and Yahr scale [51]. Control subjects reported neither a history of neurological problems nor any significant current psychiatric disorders. All participants gave their informed consent according to procedures approved by the ethics board at the University of Missouri-Columbia (Approval number: 1170557). 

### 2.2. Sample Size Justification

Central hypotheses 1 and 2 were the focus of power analysis to justify sample size in the present study. A priori sample size calculation was conducted using GPower3.1 [52] to achieve a power (1-β; Type II error rate) of 0.95 for comparing PD and controls in 2 memory experiments (3 conditions for Experiment 1 and 5 conditions for Experiment 2).

Based upon data from a relevant previous study [28], the mean effect size (d) for group comparisons was expected to be 0.83. With a multivariate analysis of variance design of 2 groups (PD vs. controls) with 3 (Experiment 1) or 5 (Experiment 1) outcome variables, a minimum total number of 30 subjects will give 95% power to detect the group differences at a significance level of α (type I error rate) = 0.05.

### 2.3. Stimuli and Procedures for Neuropsychological Experiments

Stimulus arrays were presented within a 4 × 7.3° rectangular region centered at fixation on a dark background. Arrays consisted of either two or five colored rectangles. Item positions were randomized across trials. Both red and green rectangles subtended 0.65 × 1.15° of visual angle, with orientations selected randomly from a set of four possible values (vertical, horizontal, left-tilting 45°, and right-tilting 45°). 

**Figure 1:**
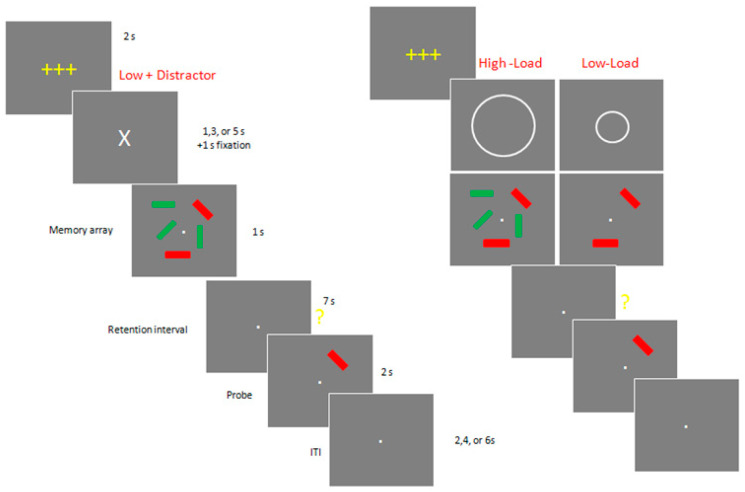
Example of a typical trial in Experiment 1. ITI: intertrial interval. +++: a get-ready signal; X: Low Load + Distractor condition; o: Low Load condition; O: High Load condition; red bars: to-be-remembered items; green bars: to-be-ignored items.

In Experiment 1 (Figure 1), each trial began with a 2 s get-ready signal (3 yellow crosses). Next, there was an instructional cue, which was followed by a 1 s long memory array, consisting of either two red or two red and three green rectangles. The instructional cue indicated whether subjects should ignore the green rectangles as distractors (“**X**”: Low Load+ Distractors) or remember them as part of target memory array with no distractors (“**o**”: Low Load or “**O**”: High Load). 

After a 7 s long retention interval, subjects were presented with a single probe stimulus for 2 s and asked to press a specified button on either the left- or right-hand keypad to report whether the orientation of the tested rectangle changed (same or different). Following an intertrial interval of 2, 4, or 6 s, the next trial commenced, starting with the yellow get-ready signal. Accuracy was emphasized over speed, and subjects were allowed to correct their response before the next trial began. Experiment 1 consists of 12 blocks of 12 trials (total 144 trials). Between blocks, participants were allowed to take as long a break as they wanted. 

**Figure 2:**
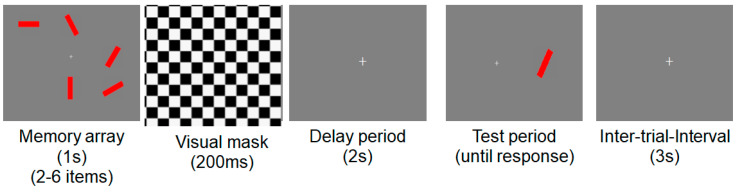
Example of a typical trial in Experiment 2. Red bars: to-be-remembered items.

In Experiment 2 (Figure 2), subjects performed a change detection task to estimate their working memory capacity. Subjects sat upright in a comfortable chair and viewed the stimuli at a distance of ~70 cm. In this version of the task, there were neither precues nor green distractors, and only relevant items (e.g., red rectangles) were presented. The number of to-be-remembered red rectangles varied from 2 to a maximum of 6, which slightly exceeds the typical capacity of an older adult. Each trial began with a 2 s get-ready signal followed by a 1 s long memory array. After a brief (200 ms) pattern mask and a 2 s delay period, the test stimulus was presented until a response was made. Following a 3 s intertrial interval, the next trial commenced. Accuracy was emphasized over speed, and subjects were allowed to correct their response before the next trial began. This version of the task was structured as 5 blocks of 32 trials (total 160 trials). 

### 2.4. MRI Image Acquisition and Image Processing 

Images were acquired on the 3-Tesla Siemens scanner at the University of Missouri’s Brain Imaging Center. Technical parameters for the structural scans were as follows: T1-weighted MPRAGE images: repetition time (TR) = 1920 ms, echo time (TE) = 2.92 ms, flip angle = 9°, field of view (FOV) = 256 mm, matrix: 256 × 256, 176 slices in the sagittal plane, voxel size = 1 × 1 × 1 mm, and slice thickness = 1 mm with acquisition time of 8 min 13 s. T2-weighted images: TR = 3200 ms, TE = 402 ms, FOV = 256 mm, matrix = 258 × 256, and slice thickness = 1 mm. 

#### 2.4.1. Brain Regions of Interest

**Figure 3:**
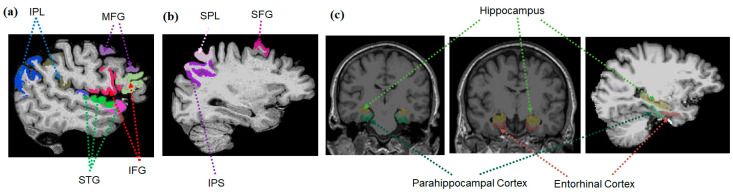
Brain regions of interests (ROIs): Brain areas related to the ventral (**a**) and dorsal (**b**) attentional systems adapted from Nani, Andrea et al. (2019) [22]. Medial temporal ROIs (**c**). IPL: inferior parietal lobe; MFG: middle frontal gyrus; STG: superior temporal gyrus; IFG: inferior frontal gyrus; SPL: superior parietal lobe; SFG: superior frontal gyrus; IPS: intraparietal sulcus.

Brain regions previously reported to be associated with attention, including attentional filtering processes (superior frontal gyrus, superior parietal lobe, and intraparietal sulcus for the dorsal attentional system and inferior and middle frontal gyri, inferior parietal lobe, and superior temporal gyrus for ventral attentional system; Figure 3a,b), working memory storage capacity during maintenance period (e.g., intraparietal sulcus; Figure 3b), or episodic memory (medial temporal lobe (hippocampus and entorhinal and parahippocampal cortices; Figure 3c)), were selected as regions of interest (ROIs). The ROIs were defined for each subject using Freesurfer. The segmentation quality was then confirmed visually by a reviewer blinded to group assignment. 

#### 2.4.2. Hippocampal Volumes and Cortical Thickness

Volumetric segmentation and cortical parcellation for thickness calculation were performed with the Freesurfer image analysis suite (http://surfer.nmr.mgh.harvard.edu/). The processing included motion correction, removal of nonbrain tissue using a hybrid watershed/surface deformation procedure [53], automated Talairach transformation, and segmentation of the deep gray matter volumetric structures and parcellation of cortical gray matter structures [54,55].

### 2.5. Statistical Analysis

Group comparisons of demographic data were conducted using one-way analysis of variance (ANOVA) or χ^2^ test. Group comparisons of neuropsychological and MRI structural ROI metrics were conducted using multivariate analysis of covariance (MANCOVA) in order to account for potential intercorrelations among outcome variables. For MANCOVA, age and education were used as covariates. To compare the performance differences between Low-Load and Low-Load with Distractor conditions, within-subjects analysis of covariance (ANCOVA) was performed using age and education as covariates. When comparing the hippocampal volume, total intracranial volume (TIV) was additionally used as a covariate. The primary neuropsychological metrics were *K* scores that were derived from hit rate (proportion of correct responses when a change was present) and false alarm rate (proportion of incorrect responses on no-change trials): *K* = *N* × (*H* − *FA*), where *N* is the number of relevant, to-be-stored items, *H* is the hit rate, and *FA* is the false alarm rate [56]. 

Association analyses of MRI structure (volume and cortical thickness) with *K* scores were conducted for controls and PD patients separately using Pearson partial correlation analyses with adjustment for age and education. Following the association analyses, regression analyses were conducted for the variables that demonstrated significant associations between MRI structural and neuropsychological metrics after controlling for age and education. In order to determine structural metrics that could explain the variances of group differences in memory metrics, a stepwise regression analysis was conducted for controls and PD separately. For the stepwise regression analysis, structural metrics that showed significant associations with memory metrics were included in addition to age and education. Statistical significance was defined as α = 0.05. The association analyses were corrected for multiple comparisons using the Holm–Bonferroni stepdown method [57] to control the familywise error rate (*FWER*) at *p* = 0.05. We report uncorrected raw *p* values but indicate significant results with *FWER*-correction. SAS 9.4 was used for all statistical analyses.

## 3. Results

### 3.1. Demographics

There were no significant group differences in age, gender, education, MMSE, and depression scores (*p* > 0.073; Table 1).

### 3.2. Group Comparison of Memory Metrics

**Figure 4:**
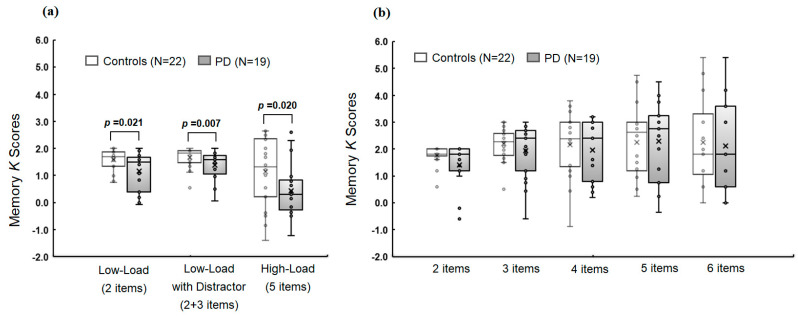
Mean *K* scores for controls and PD patients in Experiment 1 (**a**) and Experiment 2 (**b**) depending on different memory load conditions: *K = N* × (*H* − *FA*), where *N* is the number of relevant, to-be-stored items, *H* is the hit rate, and *FA* is the false alarm rate.

In Experiment 1, there were significant group differences in memory *K* scores in overall memory conditions (F(3,35) = 3.04 and *p* = 0.042) and in each individual memory condition (F(1,37) = 5.79, *p* = 0.021, and R^2^ = 0.152 for Low-Load; F(1,37) = 8.33, *p* = 0.007, and R^2^ = 0.206 for Low-Load with Distractor; F(1,37) = 5.89, *p* = 0.020, and R^2^ = 0.164 for High-Load; Figure 4a). The *K* score in the Low-Load with Distractor condition was significantly lower compared with that in Low-Load condition for PD (t = −2.81 and *p* = 0.008) but not for controls (t = −1.29 and *p* = 0.206).

In Experiment 2, there were no significant group differences in overall working memory conditions (F(5,33) = 0.84 and *p* = 0.534) or in each individual condition with different memory set sizes (F > 0.49, *p* > 0.060, and R < 0.199; to R^2^ < 0.199; Figure 4b).

### 3.3. Group Comparison of MRI Structural Metrics

There were no overall significant group differences in ROIs related to dorsal and ventral attentional systems (*p* > 0.121). When considering attention-related individual subregions, there was a significantly lower thickness in the left superior temporal gyrus for PD compared with controls (F(3,37) = 5.92, *p* = 0.020, and R^2^ = 0.270). There were no significant group differences in bilateral intraparietal sulci (*p* > 0.064) or the entorhinal and parahippocampal cortices (*p* > 0.390). There also were no significant group differences in bilateral hippocampal volumes (*p* > 0.114). 

### 3.4. Associations of MRI Structural Metrics with Memory Metrics

Within the controls, higher thickness in the left superior frontal gyrus was associated with higher memory *K* scores in the Low-Load condition of Experiment 1 (R = 0.466 and *p* = 0.038). There were no significant correlations between structural metrics in other ROIs and memory *K* scores in Experiment 1 (*p* > 0.083). 

For working memory load conditions in Experiment 2, higher thickness in the left inferior frontal opercular gyrus and lower thickness in the left superior temporal gyrus were associated with higher *K* scores in the memory load condition of 2 items (*p* < 0.048). Higher thickness in bilateral superior frontal gyri were associated with higher memory *K* scores in the memory load condition of 6 items (R > 0.460 and *p* < 0.041). None of the associations, however, remained significant after *FWER*-correction. There were no significant correlations between structural metrics in other ROIs and memory *K* scores in experiment 1 and 2 (*p* > 0.052; see Supplementary Materials Table S1a for details). 

Subsequent regression analyses confirmed that higher thickness in the left superior frontal gyrus was a significant predictor of higher *K* scores in the Low-Load condition of Experiment 1 after controlling for age and education (ß = 2.0199, t = 2.23, *p* = 0.038, and R^2^ = 0.273). Higher thickness in the left inferior frontal opercular gyrus and lower thickness in the left superior temporal gyrus were significant predictors of the memory load of 2 items in Experiment 2 (ß = 1.2427, t = 2.93, and *p* = 0.009 for inferior frontal gyrus and ß = −1.1137, t = −2.76, and *p* = 0.014 for superior temporal gyrus; total R^2^ = 0.486). 

**Figure 5:**
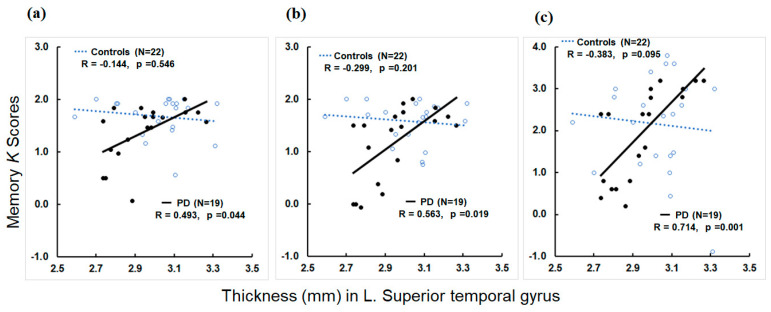
Scatter plots show memory *K* scores (*y*-axis) in Low-Load (**a**), Low-Load with Distractor (**b**), and the working memory load of 4 items (**c**) versus thickness values (mm) in the left superior temporal gyrus (*x*-axis) for controls and PD. Blue dots: controls; black dots: PD. *K = N* × (*H* − *FA*), where *N* is the number of relevant, to-be-stored items, *H* is the hit rate, and *FA* is the false alarm rate.

Within PD patients, greater thickness in the left superior temporal gyrus was associated with higher *K* scores in the Low-Load and Low-Load with Distractor conditions of Experiment 1 (R = 0.563 and *p* = 0.019 for Low-Load and R = 0.493 and *p* = 0.044 for Low-Load with Distractor; Figure 5a,b). Higher thickness in the right intraparietal sulcus was associated with higher *K* scores in the Low-Load condition (R = 0.490 and *p* = 0.046). 

For memory conditions in Experiment 2, greater thickness in the left superior temporal gyrus was associated with higher *K* scores in all the memory load conditions of 2 to 6 items (R > 0.575 and *p* < 0.016; Figure 5c for 4 items). The correlations between left superior temporal thickness and *K* scores in 3 and 4 items conditions remained significant after *FWER*-correction. Greater thickness in the right superior temporal gyrus was also associated with higher *K* scores in the memory load condition of 2 items (R = 0.514 and *p* = 0.035). Higher thickness in the left inferior frontal opercular gyrus was associated with higher *K* scores in the memory load conditions of 2, 3, 4, and 6 items (R > 0.489 and *p* < 0.046). The association between the left inferior frontal opercular gyrus and the memory load of 2 items remained significant after *FWER*-correction. Higher thickness in the right inferior frontal opercular gyrus also was associated with higher *K* scores in the memory load condition of 6 items (R = 0.681 and *p* = 0.003) that remained significant after *FWER*-correction. Higher thickness in the left inferior frontal triangular gyrus was associated with higher *K* scores in the memory load conditions of 2 to 3 items (R > 0.489 and *p* < 0.046). Higher thickness in the bilateral supramarginal gyrus was associated with higher *K* scores of the memory load condition of 6 items (R > 0.570 and *p* < 0.017). The association between the left supramarginal thickness and memory load of 6 items remained significant after *FWER*-correction. Greater left intraparietal sulcus thickness was associated with higher *K* scores of the memory load condition of 3 items (R = 0.568 and *p* = 0.017). Higher thickness in the right superior frontal gyrus was associated with higher *K* scores of the memory load conditions of 2 and 3 items (R > 0.550, *p* < 0.022). Greater thickness in the right superior parietal gyrus was associated with higher *K* scores in the memory load conditions of 2 and 3 items (R > 0.568 and *p* < 0.017). Higher thickness in the right intraparietal sulcus was associated with higher *K* scores of the memory load conditions of 3 and 6 items (R > 0.569 and *p* < 0.017). The correlations of right superior frontal and parietal gyri and intraparietal sulcus thickness with *K* scores in the memory load condition of 3 items remained significant after *FWER*-correction. 

For hippocampal volume metrics, there were no significant correlations of hippocampal volumes with any memory metrics in both Experiments 1 and 2 (*p* > 0.235; see Supplementary Materials Table S1b for details). 

Subsequent regression analyses revealed that higher thickness in the left superior temporal gyrus was a significant predictor for higher *K* scores of the Low-Load with Distractor condition in Experiment 1 (ß = 2.5189, *p* = 0.019, and R^2^ = 0.397) and of the memory load conditions of 4 and 5 items in Experiment 2 (ß = 4.1656, *p* = 0.017, and R^2^ = 0.655 for 4 items and ß = 4.4272, *p* = 0.012, and R^2^ = 0.580 for 5 items). Higher thickness in the left supramarginal gyrus significantly predicted higher *K* scores in the memory load condition of 6 items (ß = 6.6444, *p* = 0.025, and R^2^ = 0.891).

### 3.5. Stepwise Regression Analysis to Determine Factors Predicting Memory Metrics

The stepwise regression analyses were conducted using structural metrics that showed significant associations with memory metrics. In the controls, left inferior frontal-opercular and superior temporal gyri and bilateral superior frontal gyri thickness values were used for stepwise regression analyses in addition to age and education as predictors for *K* scores of Low-Load condition in Experiment 1 and the memory load conditions of 2 and 6 items in Experiment 2 (see Supplementary Materials Table S1a for association analysis results). For PD, thickness values in left inferior frontal triangular and right superior frontal and parietal gyri, as well as the bilateral inferior frontal opercular, supramarginal, superior temporal gyri, and bilateral intraparietal sulci, were used as predictors in addition to age and education.

Results revealed that in controls, higher thickness in the left superior frontal gyrus was a significant predictor for higher *K* scores of the Low-Load condition in Experiment 1 (ß = 2.0199, *p* = 0.038, and partial R^2^ = 0.202). Higher thickness in the left inferior frontal opercular gyrus and lower thickness in the left superior temporal gyrus were significant predictors for *K* scores of memory load condition of 2 items in Experiment 2 (ß = 1.2427, *p* = 0.009, and R^2^ = 0.260 for inferior frontal gyrus and ß = −1.1137, *p* = 0.014, and R^2^ = 0.229 for superior temporal gyrus). Higher thickness in the right superior frontal gyrus was a significant predictor for higher *K* scores of the memory load condition of 6 items (ß = 7.3819, *p* = 0.021, and R^2^ = 0.218).

In PD, higher thickness in the left superior temporal gyrus was a significant predictor for higher *K* scores of Low-Load (ß = 1.6034, *p* = 0.044, and R^2^ = 0.203) and Low-Load with Distractor (ß = 2.5189, *p* = 0.019, and R^2^ = 0.279) conditions in Experiment 1. Higher thickness in the left inferior frontal opercular gyrus was a significant predictor for higher *K* scores of the memory condition of 2 items in Experiment 2 (ß = 3.2703, *p* = 0.006, and R^2^ = 0.365). Higher thickness in the right superior parietal thickness was a significant predictor for higher *K* scores of the memory condition of 3 items (ß = 4.8596, *p* = 0.007, and R^2^ = 0.315). Higher thickness in the left superior temporal gyrus was a significant predictor for higher *K* scores of the memory load conditions of 4 items (ß = 4.3709, *p* = 0.001, and R^2^ = 0.360) and 5 items (ß = 4.4272, *p* = 0.012, and R^2^ = 0.228). Higher thicknesses in the right inferior frontal-opercular and supramarginal gyri were significant predictors for higher *K* scores of the memory condition of 6 items (ß = 4.7980, *p* = 0.019, and R^2^ = 0.242 for inferior frontal and ß = 6.2697, *p* = 0.019, and R^2^ = 0.093 for supramarginal).

## 4. Discussion

The present study examined mechanisms underlying memory deficits in PD and their associations with brain structural metrics. Compared to controns, PD patients had lower delayed memory scores, whereas there were no group differences in working memory performance for any memory load conditions. In addition, PD patients had lower cortical thickness in the left superior temporal gyrus, a region related to the ventral attentional system. To the contrary, there were no volume or cortical thickness differences in ROIs related to the dorsal attentional system, working memory storage capacity, or episodic memory. Moreover, lower cortical thickness values in the left superior temporal gyrus were significant predictors of lower delayed memory scores in Low-Load and Low-Load with Distractor conditions and lower working memory scores in the memory load conditions of 4 and 5 items. The present findings suggest that memory deficits in PD may partly be due to impaired attentional filtering of unnecessary information from memory space and memory consolidation processes that may be related to superior temporal neurodegeneration. The current findings have clinical implications for the development of effective treatment strategies to delay progression of cognitive deterioration in PD.

### 4.1. Memory Deficits in PD Due to Impaired Memory Consolidation Process

In the present study, two different delay periods (2 s vs. 7 s) were utilized between memory and test arrays to mimic working memory and episodic memory formation processes. Interestingly, PD patients’ memory scores were comparable to those of controls when they were tested 2 s after the memory array offset, whereas their memory performance became significantly worse with a 7 s delay period. This result suggests that PD patients’ ability to encode and retrieve visual information may be comparable to that of controls. In addition, PD patients may also have comparable information maintenance ability compared to controls, at least for a short amount of time, suggestive of intact working memory storage capacity. These findings are inconsistent with a previous finding demonstrating that PD patients had lower memory scores and CDA (contralateral delay activity) amplitudes (EEG correlates reflecting items held in working memory) in memory load conditions, even with no distractors [28]. In that study, however, a bilateral display was utilized to measure CDA, which required additional filtering by the participants, even in conditions with no distractors. So, it is possible that patients’ lower memory scores and CDA amplitudes in that study could be due to impaired filtering rather than diminished working memory storage capacity. The current finding supports this interpretation. 

Instead, PD patients seemed to have difficulty with continuously holding information in memory over a prolonged period, where memory consolidation may increasingly gain importance to form stable episodic memory. Memory consolidation is a process by which a temporary and unstable memory trace is transformed into a more stable and long-lasting memory [58] and may serve as a critical component for successful episodic memory formation process. Note that previous studies that reported impaired episodic memory performance in PD typically tested memory performance after a ~20 min delay period by utilizing standard neuropsychological test batteries [41,42]. The current findings, however, suggest that impaired episodic memory formation processes may be detected as early as within a 7 s delay period. Thus, the current finding of lower delayed memory performance in PD without difference in working memory performance suggests intact working memory storage capacity but an impaired memory consolidation process in PD. The present results are in line with previous findings reporting inability to learn new information over time among PD patients [48,49,59]. Note that memory consolidation ability is crucial to learn information over time.

Given that the major and early AD-related behavioral deficits entail episodic memory decline [36,60], the current finding of impaired memory consolidation in PD suggests that AD-related early neurobehavioral changes can occur in PD with still intact working memory storage capacity. 

### 4.2. Memory Deficits in PD Due to Impaired Attentional Filtering

Both controls and PD patients had some difficulty ignoring distractors during encoding of information into memory. They demonstrated lower memory scores in the Low-Load with Distractor compared with the Low-Load condition, although the number of to-be-remembered items was the same for both conditions with and without distractors. Interestingly, the memory score difference between these two memory load conditions was significant only for PD patients, suggesting that lower memory scores in PD are partly due to attentional filtering deficits. This finding is consistent with a previous study [28] reporting that PD patients had lower memory scores but higher CDA (contralateral delay activity) amplitudes in memory load conditions with distractors, suggestive of unnecessary storage for task-irrelevant items [21]. Recent evidence also suggests that PD patients particularly had difficulty with selectively updating relevant information into memory, while their ability simply to hold information in working memory was comparable to that of the controls [30]. 

The current result is in line with previous findings reporting a critical role of the frontostriatal pathway in controlling the access of incoming information into memory systems [20,61,62]. Patients with impaired frontostriatal pathways may be especially vulnerable to these filtering deficits. Thus, the loss of dopaminergic input to the basal ganglia in PD may lead to diminished frontostriatal functions and reduced ability to filter out distractors, so that they unnecessarily usurp memory space with irrelevant information. Indeed, PD patients seem to be vulnerable to filtering deficits in a general sense, as such PD patients have more difficulty with inhibiting automatic responses to to-be-ignored salient but irrelevant stimuli, such as flanking distractors [63,64,65]. 

The current findings have important clinical relevance not only for a better understanding of the exact nature of memory deficits in PD but also for developing effective treatment strategies to improve memory in PD. For example, cognitive training focusing on attentional filtering may be useful to improve memory in PD. Recent evidence supports this idea by reporting that memory training focusing on updating old irrelevant information with newer relevant information into memory not only improved memory performance but also goal-directed motor task performance, as well as increased brain activation in the frontostriatal pathway [66,67].

Several factors may have co-influenced cognitive decline in PD, one of which can be depressive status [30]. Clinical depression is fairly common in PD, comprising about 40–50% of PD patients [68]. Similar brain networks (e.g., frontostriatal pathway) seem to be involved in both cognitive and affective control processes [69,70]. The current findings of lower delayed memory scores throughout all memory load conditions, however, cannot be ascribed to PD patients’ depressive status, because our PD patients had comparable depression scores to those of the controls. It is worthwhile to mention that there was one PD patient who had a GDS score of 11 out of 30, indicative of mild depression. The pattern of the results, however, remained the same with and without this patient’s data in the analysis (data not shown). 

### 4.3. Neural Correlates of Memory Deficits in PD

In the present study, we examined brain structural metrics (volumes and cortical thickness) in areas that are known to be related to attentional processes, including attentional filtering, working memory storage capacity, and episodic memory, including memory consolidation processes. Consistent with comparable working memory storage capacity throughout different memory load conditions, there were no significant group differences in the intraparietal sulcus, an area known to be sensitive to working memory storage capacity [71,72,73]. Instead, PD patients had lower cortical thickness in the left superior temporal gyrus. Moreover, lower thickness values in this area significantly predicted lower memory scores in both delayed and working memory tasks. It is worthwhile to note that recent studies reported that electrical stimulations to the lateral temporal cortex, including middle and superior temporal gyri, during the information encoding stage improved episodic memory performance in human subjects more than when the stimulations were applied to nonlateral temporal areas, including medial temporal lobe areas, supporting superior temporal involvement in episodic memory formation [74,75]. 

Given the close relationship between medial temporal areas and episodic memory, previous brain stimulation studies in human subjects primarily utilized medial temporal sites (e.g., entorhinal cortex and hippocampus) to improve memory. The findings were, however, inconsistent [76,77], demonstrating even impaired episodic memory performance following electrical stimulation [77,78]. Thus, the current finding of the superior temporal involvement in memory deficits in PD along with previous brain stimulation studies have important clinical implications for developing novel target sites of brain stimulation to improve memory in PD and other AD at-risk populations [79,80]. 

The superior temporal gyrus is generally known to be part of ventral attention network that is generally associated with the bottom-up detection of salient stimuli by shifting attention to unexpected information, while a dorsal attention network employs top-down goal-directed attentional shifts to designated features of stimuli [22]. Both ventral and dorsal attention networks, however, seem to simultaneously influence and integrate each other in real-life situations [22]. The volume of the left superior temporal gyrus has also been reported to sensitively reflect verbal working memory capacity and ability to comprehend spoken sentences [81,82]. Since properly understanding spoken sentences may be involved in accessing to long-term storage of lexico-semantic representations of verbal information, the role of the left superior temporal gyrus in accessing long-term storage, especially for verbal information, is implicated [81]. The significant associations of the left superior temporal gyrus thickness with memory capacity scores in both delayed and working memory conditions observed in the present study may extend previous findings by suggesting that the role of the left superior temporal gyrus may be multimodal and generic to working memory capacity, as well as transferring information to and accessing long-term memory storage. Future studies are warranted to replicate the current findings and test this intriguing hypothesis. 

It is intriguing to note that there were no volumetric or thickness differences in any of medial temporal ROIs or associations with memory metrics, although PD patients demonstrated robust decline in delayed memory performance. It is possible that our sample size was too small to reliably detect structural differences in medial temporal areas. It also is possible that patients’ lower delayed memory performance observed in this study may partly be associated with early brain microstructural or functional changes that may occur before medial temporal morphological changes [83]. For example, previous studies reported that diffusion tensor imaging (DTI) metrics that measure random translational water motion may reflect early microstructural changes [84]. These DTI measures have been suggested to be more sensitive than volume or thickness measures in capturing AD-related early brain changes, including changes in the medial temporal lobe [85,86,87,88,89]. Further studies utilizing multimodal brain imaging markers that sensitively capture early brain microstructural or functional changes should be warranted to confirm the current findings and to elucidate neural correlates of delayed memory deficits in PD. 

## 5. Limitations and Conclusions

The is the first study reporting that impaired attentional filtering and memory consolidation problems may contribute to memory deficits in PD and that superior temporal neurodegeneration may partly underlie these impaired cognitive processes. There are several limitations to this study. First, the sample sizes were relatively small to generalize the finding. A larger sample size is needed to strengthen and increase the validity of the current findings. Second, levodopa equivalent daily dose (LEDD) information was not acquired for the PD group, although information about types of PD medication was collected and reported. This can limit the assessment of disease severity and potential relationship between LEDD and the degree of memory deficits in PD. Nevertheless, the current findings can contribute to better management of PD’s cognitive symptoms by providing guidelines for the development of effective treatment strategies (e.g., effective filtering training and novel brain stimulation target site for episodic memory improvement) to delay the progression of cognitive deterioration in PD.

## Figures and Tables

**Table 1:** Demographics.

	Controls (N = 22)	PD Patients (N = 19)	*p*-Values
Age (y)	69.05 ± 5.58	66.16 ± 8.81	0.211
Gender (m/f)	12/10	14/5	0.205
Education (years)	14.77 ± 3.19	16.63 ± 3.25	0.073
MMSE	29.40 ± 0.99	29.00 ± 1.29	0.284
Hoehn and Yahr Scale (1/2/3)	0	2.03 ± 0.77 (7/8/4)	
Disease duration (years)	0	6.65 ± 4.76	
GDS	2.78 ± 2.17	4.11 ± 3.11	0.147

Note. Descriptive data for participants’ demographics: MMSE: Mini-Mental State Exam; GDS: Geriatric Depression Scale.

## Data Availability

Data can be available upon request.

## Supplementary Materials

The following supporting information can be downloaded at: https://www.mdpi.com/article/10.3390/jcm12144594/s1, Table S1: Association analyses of MRI structural metrics with memory K scores for controls and PD patients.
