# Supplementary material for: Memory Deficits in Parkinson’s Disease Are Associated with Impaired Attentional Filtering and Memory Consolidation Processes
Source: J Clin Med. 2023 Jul 10;12(14):4594. doi: 10.3390/jcm12144594 (PMC10380592; doi:10.3390/jcm12144594)
Supplement: Supplementary file 1 [file jcm-12-04594-s001.zip › jcm-2376968-supplementary.pdf]

Journal of
*Clinical Medicine*

MDPI

**Table S1.** Association analyses of MRI structural metrics with memory K scores for controls and PD patients .

**a-1. Controls (N=22)**
*Correlation coefficient R*
*(p-value)*

| | Delayed Memory | | | Working Memory | | | | |
|---|---|---|---|---|---|---|---|---|
| | Low-Load | Low-Load with Distrac-tor | High-Load | Memory Load 2 | Memory Load 3 | Memory Load 4 | Memory Load 5 | Memory Load 6 |
| **Areas related to the ventral attentional system** | | | | | | | | |
| L. inferior Frontal-Opercular | 0.372 (0.107) | -0.017 (0.944) | -0.062 (0.794) | **0.481 (0.032)** | 0.208 (0.380) | 0.314 (0.178) | 0.022 (0.928) | 0.067 (0.780) |
| L. Inferior Frontal-Triangular | 0.222 (0.348) | -0.177 (0.456) | 0.122 (0.607) | 0.440 (0.052) | 0.362 (0.117) | 0.365 (0.114) | 0.025 (0.916) | 0.123 (0.606) |
| L. middle Frontal Gyrus | 0.254 (0.279) | -0.34 (0.887) | 0.-99 (0.679) | 0.076 (0.752) | -0.084 (0.725) | 0.052 (0.828) | -0.099 (0.677) | 0.190 (0.423) |
| L. Angular Gyrus | 0.190 (0.422) | 0.068 (0.776) | 0.258 (0.271) | -0.072 (0.762) | -0.049 (0.838) | -0.013 (0.955) | 0.110 (0.644) | 0.306 (0.190) |
| L. Supra-marginal gyrus | 0.149 (0.531) | -0.094 (0.693) | -0.011 (0.964) | 0.064 (0.790) | -0.119 (0.618) | 0.025 (0.918) | -0.094 (0.693) | -0.079 (0.740) |
| L. superior Temporal gyrus | -0.144 (0.546) | -0.299 (0.201) | -0.311 (0.181) | **-0.448 (0.048)** | -0.359 (0.120) | -0.383 (0.095) | -0.121 (0.613) | -0.009 (0.969) |
| **Areas related to the dorsal attentional system and working memory storage capacity** | | | | | | | | |
| L. superior Frontal gyrus | **0.466 (0.038)** | 0.148 (0.532) | 0.070 (0.770) | 0.088 (0.711) | 0.160 (0.499) | 0.221 (0.350) | 0.107 (0.653) | **0.460 (0.041)** |
| L. superior Parietal Gyrus | 0.292 (0.212) | 0.098 (0.680) | 0.020 (0.935) | -0.071 (0.765) | 0.021 (0.930) | -0.008 (0.975) | -0.223 (0.346) | 0.050 (0.835) |
| L. Intra-parietal Sulcus | 0.346 (0.135) | 0.133 (0.577) | -0.052 (0.829) | -0.214 (0.364) | 0.004 (0.988) | -0.022 (0.926) | -0.101 0.673) | 0.092 (0.701) |
| **Areas related to episodic memory** | | | | | | | | |
| L. Entorhinal Cortex | -0.021 (0.929) | 0.155 (0.514) | 0.246 (0.296) | 0.315 (0.176) | 0.429 (0.059) | 0.332 (0.152) | 0.265 (0.259) | 0.255 (0.278) |
| L. Para-hippocam-pal Gyrus | 0.027 (0.911) | 0.034 (0.887) | 0.057 (0.810) | -0.045 (0.851) | -0.138 (0.561) | -0.181 (0.44 | -0.253 (0.283) | -0.351 (0.129) |
| L. hippocampal volume | 0.118 (0.622) | 0.275 (0.240) | 0.166 (0.483) | 0.366 (0.113) | 0.155 (0.514) | 0.350 (0.131) | 0.183 (0.441) | 0.030 (0.900) |

| a-2. Controls (N=22)<br>*Correlation coefficient R*<br>*(p-value)* | | | | | | | | |
|---|---|---|---|---|---|---|---|---|
| | Delayed Memory | | | Working Memory | | | | |
| | Low-Load | Low-Load with Distrac-tor | High-Load | Memory Load 2 | Memory Load 3 | Memory Load 4 | Memory Load 5 | Memory Load 6 |
| Areas related to the ventral attentional system | | | | | | | | |
| R. inferior Frontal-Opercular | 0.224<br>(0.343) | 0.109<br>(0.648) | 0.104<br>(0.663) | 0.198<br>(0.403) | -0.088<br>(0.712) | 0.123<br>(0.605) | -0.140<br>(0.556) | -0.052<br>(0.829) |
| R. Inferior Frontal-Triangular | 0.327<br>(0.160) | 0.054<br>(0.823) | -0.042<br>(0.860) | -0.218<br>(0.355) | -0.098<br>(0.680) | -0.180<br>(0.448) | -0.249<br>(0.289) | -0.084<br>(0.726) |
| R. middle Frontal Gyrus | 0.397<br>(0.083) | 0.239<br>(0.311) | 0.242<br>(0.303) | -0.045<br>(0.850) | 0.054<br>(0.820) | 0.191<br>(0.421) | 0.085<br>(0.722) | 0.256<br>(0.277) |
| R. Angular Gyrus | 0.084<br>(0.725) | -0.041<br>(0.864) | -0.028<br>(0.906) | -0.116<br>(0.627) | 0.048<br>(0.840) | -0.070<br>(0.770) | 0.092<br>(0.701) | 0.190<br>(0.422) |
| R. Supra-marginal gyrus | 0.319<br>(0.171) | 0.101<br>(0.672) | -0.117<br>(0.623) | -0.232<br>(0.325) | -0.075<br>(0.755) | -0.264<br>(0.261) | -0.320<br>(0.169) | -0.105<br>(0.659) |
| R. superior Temporal gyrus | 0.281<br>(0.231) | 0.051<br>(0.831) | -0.005<br>(0.982) | -0.235<br>(0.319) | -0.3005<br>(0.198) | -0.253<br>(0.2883) | -0.328<br>(0.158) | -0.155<br>(0.515) |
| Areas related to the dorsal attentional system and working memory storage capacity | | | | | | | | |
| R. superior Frontal gyrus | 0.324<br>(0.164) | 0.108<br>(0.649) | 0.034<br>(0.886) | 0.007<br>(0.975) | 0.121<br>(0.613) | 0.109<br>(0.648) | 0.235<br>(0.318) | **0.512**<br>**(0.021)** |
| R. superior Parietal Gyrus | 0.211<br>(0.371) | 0.096<br>(0.688) | 0.115<br>(0.628) | -0.161<br>(0.498) | 0.102<br>(0.669) | -0.080<br>(0.738) | 0.037<br>(0.876) | 0.276<br>(0.239) |
| R. Intra-parietal Sulcus | 0.151<br>(0.525) | 0.024<br>(0.920) | 0.010<br>(0.965) | -0.232<br>(0.326) | -0.055<br>(0.818) | -0.129<br>(0.587) | 0.087<br>(0.717) | 0.232<br>(0.325) |
| Areas related to episodic memory | | | | | | | | |
| R. Entorhinal Cortex | 0.260<br>(0.268) | 0.058<br>(0.809) | 0.320<br>(0.170) | 0.249<br>(0.290) | 0.098<br>(0.683) | 0.261<br>(0.266) | 0.006<br>(0.982) | 0.238<br>(0.313) |
| R. Para-hippocam-pal Gyrus | 0.068<br>(0.775) | 0.030<br>(0.899) | 0.024<br>(0.922) | -0.124<br>(0.601) | -0.286<br>(0.221) | -0.145<br>(0.543) | -0.068<br>(0.774) | -0.265<br>(0.258) |
| R. hippocampal volume | 0.061<br>(0.798) | 0.144<br>(0.544) | 0.061<br>(0.798) | 0.279<br>(0.234) | -0.020<br>(0.934) | 0.255<br>(0.277) | 0.114<br>(0.632) | -0.063<br>(0.792) |

**b-1. PD Patients (N=19)**
*Correlation coefficient R*
*(p-value)*

| | Delayed Memory | | | Working Memory | | | | |
|---|---|---|---|---|---|---|---|---|
| | Low-Load | Low-Load with Distractor | High-Load | Memory Load 2 | Memory Load 3 | Memory Load 4 | Memory Load 5 | Memory Load 6 |
| Areas related to the ventral attentional system | | | | | | | | |
| L. inferior Frontal-Opercular | 0.237<br>(0.360) | 0.330<br>(0.196) | 0.255<br>(0.322) | **0.640***<br>**(0.006)** | **0.546**<br>**(0.024)** | **0.505**<br>**(0.039)** | 0.293<br>(0.254) | **0.487**<br>**(0.047)** |
| L. Inferior Frontal-Triangular | 0.206<br>(0.427) | 0.195<br>(0.453) | 0.019<br>(0.941) | **0.582**<br>**(0.014)** | **0.489**<br>**(0.046)** | 0.429<br>(0.086) | 0.173<br>(0.506) | 0.212<br>(0.414) |
| L. middle Frontal Gyrus | -0.219<br>(0.398) | -0.259<br>(0.315) | 0.133<br>(0.610) | 0.125<br>(0.634) | 0.170<br>(0.513) | 0.162<br>(0.534) | -0.050<br>(0.848) | 0.475<br>(0.054) |
| L. Angular Gyrus | 0.046<br>(0.861) | 0.161<br>(0.538) | 0.258<br>(0.318) | 0.215<br>(0.408) | 0.284<br>(0.269) | 0.023<br>(0.930) | -0.025<br>(0.923) | 0.256<br>(0.322) |
| L. Supra-marginal gyrus | 0.263<br>(0.309) | 0.232<br>(0.370) | 0.414<br>(0.099) | 0.466<br>(0.060) | 0.431<br>(0.084) | 0.208<br>(0.423) | 0.162<br>(0.535) | **0.679***<br>**(0.003)** |
| L. superior Temporal Gyrus | **0.493**<br>**(0.044)** | **0.563**<br>**(0.019)** | 0.359<br>(0.157) | **0.604***<br>**(0.010)** | **0.575**<br>**(0.016)** | **0.714***<br>**(0.001)** | **0.594**<br>**(0.012)** | **0.595**<br>**(0.012)** |
| Areas related to the dorsal attentional system and working memory storage capacity | | | | | | | | |
| L. superior Frontal gyrus | -0.112<br>(0.668) | -0.215<br>(0.407) | 0.115<br>(0.660) | 0.134<br>(0.607) | 0.203<br>(0.434) | 0.010<br>(0.969) | -0.086<br>(0.744) | -0.093<br>(0.724) |
| L. superior Parietal Gyrus | 0.188<br>(0.469) | 0.037<br>(0.887) | 0.165<br>(0.526) | 0.417<br>(0.096) | 0.464<br>(0.061) | 0.271<br>(0.293) | 0.307<br>(0.231) | 0.355<br>(0.162) |
| L. Intra-parietal Sulcus | 0.359<br>(0.157) | 0.139<br>(0.596) | 0.315<br>(0.219) | 0.325<br>(0.203) | **0.568**<br>**(0.017)** | 0.211<br>(0.417) | 0.282<br>(0.274) | 0.346<br>(0.174) |
| Areas related to episodic memory | | | | | | | | |
| L. Entorhinal Cortex | 0.012<br>(0.965) | 0.156<br>(0.550) | -0.215<br>(0.408) | 0.065<br>(0.804) | -0.197<br>(0.448) | -0.062<br>(0.812) | -0.038<br>(0.885) | -0.170<br>(0.515) |
| L. Para-hippocampal Gyrus | 0.156<br>(0.549) | 0.168<br>(0.520) | 0.363<br>(0.152) | -0.107<br>(0.683) | 0.217<br>(0.403) | 0.014<br>(0.958) | -0.112<br>(0.669) | 0.391<br>(0.121) |
| L. hippocampal volume | -0.099<br>(0.704) | -0.195<br>(0.453) | -0.246<br>(0.342) | 0.246<br>(0.342) | 0.098<br>(0.708) | -0.052<br>(0.842) | 0.021<br>(0.936) | -0.048<br>(0.856) |

**b-2. PD Patients (N=19)**
*Correlation coefficient R*
*(p-value)*

| | Delayed Memory | | | Working Memory | | | | |
|---|---|---|---|---|---|---|---|---|
| | Low-Load | Low-Load with Distractor | High-Load | Memory Load 2 | Memory Load 3 | Memory Load 4 | Memory Load 5 | Memory Load 6 |
| Areas related to the ventral attentional system | | | | | | | | |
| R. inferior Frontal-Opercular | -0.243<br>(0.348) | -0.073<br>(0.779) | 0.255<br>(0.323) | 0.262<br>(0.309) | 0.094<br>(0.719) | 0.227<br>(0.381) | 0.009<br>(0.971) | **0.681***<br>**(0.003)** |
| R. Inferior Frontal-Triangular | -0.118<br>(0.652) | -0.034<br>(0.896) | -0.288<br>(0.262) | -0.077<br>(0.768) | -0.183<br>(0.482) | -0.308<br>(0.229) | -0.421<br>(0.093) | 0.026<br>(0.920) |
| R. middle Frontal | 0.082 | 0.052 | 0.123 | 0.190 | 0.368 | 0.227 | 0.005 | 0.168 |

| | | | | | | | | |
|---|---|---|---|---|---|---|---|---|
| Gyrus | (0.754) | (0.842) | (0.637) | (0.465) | (0.147) | (0.381) | (0.984) | (0.520) |
| R. Angular Gyrus | 0.027 (0.917) | 0.217 (0.404) | -0.038 (0.884) | 0.203 (0.436) | 0.148 (0.571) | 0.086 (0.743) | -0.098 (0.709) | 0.238 (0.358) |
| R. Supra-marginal gyrus | -0.071 (0.786) | 0.149 (0.569) | 0.227 (0.381) | 0.365 (0.150) | 0.217 (0.403) | 0.240 (0.353) | 0.005 (0.984) | **0.0570 (0.017)** |
| R. superior Temporal Gyrus | -0.002 (0.993) | 0.125 (0.634) | 0.004 (0.988) | **0.514 (0.035)** | 0.279 (0.278) | 0.369 (0.145) | 0.218 (0.402) | 0.104 (0.690) |
| Areas related to the dorsal attentional system and working memory storage capacity | | | | | | | | |
| R. superior Frontal gyrus | 0.232 (0.370) | 0.253 (0.327) | 0.134 (0.608) | **0.550 (0.022)** | **0.568* (0.017)** | 0.370 (0.144) | 0.217 (0.402) | 0.107 (0.684) |
| R. superior Parietal Gyrus | 0.375 (0.138) | 0.417 (0.096) | 0.295 (0.251) | **0.568 (0.017)** | **0.630* (0.007)** | 0.386 (0.126) | 0.239 (0.356) | 0.297 (0.248) |
| R. Intra-parietal Sulcus | **0.490 (0.046)** | 0.438 (0.079) | 0.115 (0.659) | 0.442 (0.076) | **0.574* (0.016)** | 0.447 (0.072) | 0.395 (0.117) | **0.569 (0.017)** |
| Areas related to episodic memory | | | | | | | | |
| R. Entorhinal Cortex | -0.202 (0.438) | -0.004 (0.989) | -0.324 (0.204) | 0.110 (0.673) | -0.039 (0.882) | 0.017 (0.948) | -0.032 (0.902) | -0.030 (0.908) |
| R. Para-hippocampal Gyrus | -0.202 (0.436) | -0.055 (0.834) | 0.088 (0.737) | 0.025 (0.924) | 0.070 (0.788) | 0.070 (0.790) | -0.130 (0.618) | 0.219 (0.398) |
| R. hippocampal volume | -0.086 (0.744) | -0.030 (0.910) | -0.304 (0.235) | 0.289 (0.261) | 0.072 (0.784) | -0.029 (0.913) | -0.031 (0.906) | -0.091 (0.729) |

Association analysis results showing Pearson correlation coefficient R (p-value). * indicates significant results at *FWER*=0.05. Association analyses were conducted with adjustment for age and education.
